# Nuclear resonance fluorescence drug inspection

**DOI:** 10.1038/s41598-020-80079-6

**Published:** 2021-01-14

**Authors:** Haoyang Lan, Tan Song, Xingde Huang, Shengqiang Zhao, Jianliang Zhou, Zhichao Zhu, Yi Xu, Dimiter L. Balabanski, Wen Luo

**Affiliations:** 1 School of Nuclear Science and Technology, University of South China, Hengyang, 421001 China; 2 Extreme Light Infrastructure Nuclear Physics (ELI-NP), Horia Hulubei National Institute for R&D in Physics and Nuclear Engineering (IFIN-HH), 30 Reactorului Str., 077125 Buchurest-Magurele, Romania; 3 National Exemplary Base for International Sci and Tech. Collaboration of Nuclear Energy and Nuclear Safety, University of South China, Hengyang, 421001 China

**Keywords:** Characterization and analytical techniques, Nuclear physics

## Abstract

There is an increasing challenge to prevent illicit drug smuggling across borders and seaports. However, the existing techniques in-and-of-themselves are not sufficient to identify the illicit drugs rapidly and accurately. In the present study, combining nuclear resonance fluorescence (NRF) spectroscopy and the element (or isotope) ratio approach, we present a novel inspection method that can simultaneously reveal the elemental (or isotopic) composition of the illicit drugs, such as widely abused methamphetamine, cocaine, heroin, ketamine and morphine. In the NRF spectroscopy, the nuclei are excited by the induced photon beam, and measurement of the characteristic energies of the emitted $\gamma$ rays from the distinct energy levels in the excited nuclei provides “fingerprints” of the interested elements in the illicit drugs. The element ratio approach is further used to identify drug elemental composition in principle. Monte Carlo simulations show that four NRF peaks from the nuclei $^{12}$C, $^{14}$N and $^{16}$O can be detected with high significance of 7−24$\sigma$ using an induced photon beam flux of $10^{11}$. The ratio of $^{14}N$/$^{12}C$ and/or $^{16}O$/$^{12}C$ for illicit drugs inspected are then extracted using the element ratio approach. It is found that the present results of simulations are in good agreement with the theoretical calculations. The feasibility to detect the illicit drugs, inside the 15-mm-thick iron shielding, or surrounded by thin benign materials, is also discussed. It is indicated that, using the state-of-the-art $\gamma$-ray source of high intensity and energy-tunability, the proposed method has a great potential for identifying drugs and explosives in a realistic measurement time.

## Introduction

The smuggling of contraband is one of the biggest threats to public health, human welfare and national security. Currently, illicit drug trafficking is becoming more and more rampant. For instance, the global cocaine production and seizures reach record highs in 2017, reaching 1976 tons and 1275 tons, respectively^1^. There are large quantities of unseized drugs still passing readily across national borders, which presents a continual challenge to the law enforcement officials. The fight against drug smuggling at ports of entry still relies much on experienced narcotic agents and well trained sniffer dogs. So far, Raman spectroscopy^2,3^ is widely used on the forensic analysis of drugs. However, this technique is only limited to identify drug trafficking patterns and distribution networks, thus leaving behind the problem of large quantities of unseized drugs. It seems impossible to identify smuggled drugs by checking on every single parcel, air courier and maritime container. An efficient nondestructive inspection method for concealed illicit drugs is indispensable for reducing this combined danger.

Nuclear resonance fluorescence^4^ (NRF) describes the X($\gamma$,$\gamma$’)X reaction in which a photon $\gamma$ is resonantly absorbed by the nucleus X and then re-emitted as the excited nucleus subsequently transits to its lower states, particularly the ground state. The energy of NRF $\gamma$ rays depends merely on the distribution of nuclear levels, and the spectrometry of NRF $\gamma$ rays can then be used for nondestructive detection of heavily shielded materials, considering deep penetration of energetic $\gamma$ rays. This nondestructive detection could be beneficial in industrial applications^5–7^ as well as nuclear safeguards^8,9^. Bremsstrahlung photons^10^ and neutrons^11,12^ have been studied as probes for measuring composition of materials that are concealed by heavy shields. However, bremsstrahlung photons generally trigger the NRF processes in low-lying excited states (within 3 MeV), because of decreasing intensity with energy and high dose to the shielded objects, and the exact determination of neutron energy is difficult because of the multiple neutron scattering inside the objects. Recently, laser-Compton scattering (LCS) $\gamma$ rays have been proposed for nondestructive detection of special nuclear materials (SNM)^13,14^ (such as $^{208}$Pb and $^{238}$U) and chemical compounds ^15,16^. Moreover, the LCS $\gamma$-ray source can generate energy-tunable, quasi-monochromatic $\gamma$ rays^17,18^ and have been used for studying nuclear physics^19,20^ and nuclear astrophysics^21^, industrial and medical applications^22–25^.

**Figure 1:**
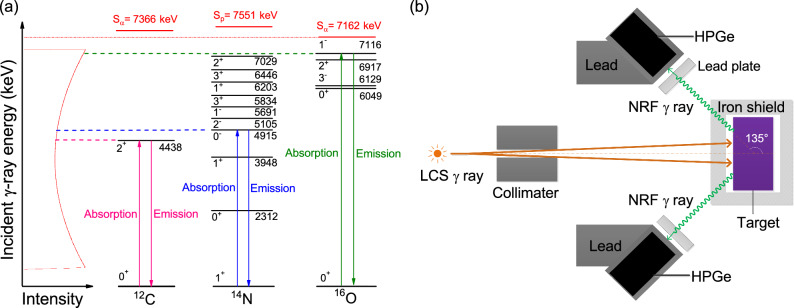
(**a**) Schematic view of nuclear excitation and de-excitation. If the energy of the $\gamma$-ray beam, which is produced by Compton backscattering of a laser pulse from high-energy electron beam, is identical with the excitation energy of the nucleus of interest, it can be effectively absorbed in the nucleus, which de-excites subsequently by emission of NRF photons. (**b**) Schematic view of the NRF-based inspection system using a LCS $\gamma$-ray source. After collimation, the LCS $\gamma$-ray beam penetrates through the iron shield and then impinges on the drug target, producing NRF $\gamma$ rays. In order to realize backscatter NRF inspection, four high-purity germanium (HPGe) detectors are assembled on a detection plane with large angle of 135$^{\circ }$ with respect to the beam propagation. The lead plates are installed in front of HPGe detectors to improve the ability to operate detectors by reducing the intensity of the low-energy portion of the scattered beam.

In this study, we investigate through Monte Carlo (MC) simulations nondestructive inspection of illicit drugs by exploiting NRF interaction in light elements with an intense LCS $\gamma$-ray source (see Fig. 1). It uses isotope-specific data signatures, which can be used to obtain a unique fingerprint of the object, to identify elemental components ($^{12}$C, $^{14}$N and $^{16}$O) of illicit drugs. Unlike fast neutrons^11^, which does not have this isotope-specificity for both low and high Z nuclei, the backscatter NRF signals can be made highly specific and sensitive to the presence and abundance of individual isotopes. Also unlike bremsstrahlung radiations, the LCS $\gamma$ rays can reduce the background flux at the low-energy side produced from atomic processes in the object^26^.

There are five widely abused drugs to be considered, including cocaine, heroin, ketamine, methamphetamine and morphine. To identify these abused drugs by their NRF signatures, we propose a novel approach of element ratio, $^{14}N/^{12}C$ and $^{16}O/^{12}C$. Results show that four NRF signals from drug components can be readily differentiated by spectrometry of NRF $\gamma$ rays, even in the presence of metal shielding. The significance of the resulting NRF peaks reaches 7−24$\sigma$ with LCS $\gamma$-ray flux of 10$^{11}$. The element ratios of $^{14}N/^{12}C$ and/or $^{16}O/^{12}C$ simulated for five abused drugs are in good agreement with the theoretical values. It hence indicates that this combined detection method can identify the illicit drugs with high confidence in a practical measurement time.

## Results

### NRF yields in backscatter inspection

**Figure 2:**
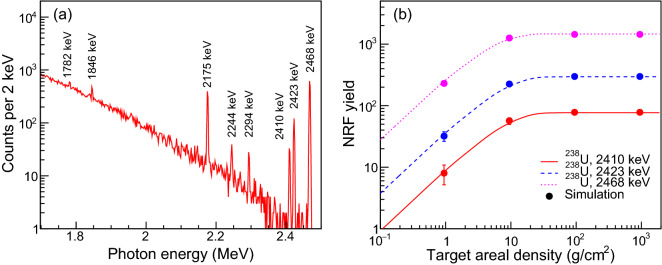
(**a**) Simulation results of energy spectrum recorded by the HPGe detectors at an incident $\gamma$ flux of $10^{11}$ photons for target areal density of 200 g/cm$^{2}$. (**b**) Count rates as a function of target areal density for three NRF lines of $^{238}$U with high energy of 2410, 2423 and 2468 keV. The curves present semi-analytical model predictions (via integration of Eq.  (1)), while the points correspond to NRF yields extracted from the energy spectrum. Here error bars denote $\pm 1 \sigma$ statistical uncertainties.

In this section, we use NRF cross section, $\sigma _{NRF}(E)$, and angular distribution, $W(\theta )$ (see Methods), to construct an semi-analytical expression for the expected NRF counts. This expression can be further used to validate the simulation algorithm (see Methods). For a collimated LCS photon beam of incident flux, *I*(*E*) interacting with the target, a small part of photon flux near the resonant energy $E_{NRF}$ will undergo resonant (NRF) and non-resonant (atomic) interactions inside the target. The resulting NRF yield then produces a double-differential rate of NRF interactions in the infinitesimal solid angle $d\Omega$^27^,

1 $$\begin{aligned} \frac{d^{2}Y}{dE d\Omega }= I(E) \mu _{NRF}(E) \frac{W(\theta )}{4\pi } \frac{1 - exp\left[ - L \mu _{eff}(E,E',\theta ) \right] }{\mu _{eff}(E,E',\theta )} \epsilon (E'), \end{aligned}$$

where *E* is the energy of primary LCS photons. $E' = E - E_{rec}$ is the energy of photons induced by NRF. $\theta$ is the emission angle of the NRF photons relative to the beam propagation, *L* is the thickness of the irradiated target, $\epsilon (E')$ is the intrinsic detection efficiency for photopeak, $\mu _{NRF}(E) = N\sigma _{NRF}(E)$ denotes the linear attenuation coefficient with *N* being the target number density, and $\mu _{eff}(E,E',\theta )$ is the effective attenuation coefficient, which is given by

2 $$\begin{aligned} \mu _{eff}(E,E',\theta ) = \mu _{NRF}(E) + \mu _{nr}(E) + \frac{\mu _{nr}(E')}{cos\theta }. \end{aligned}$$

Here $\mu _{nr}(E)$ and $\mu _{nr}(E')$ are the non-resonant attenuation coefficient for the primary LCS photons and secondary photons, respectively.

In this study, the backscatter inspection setup is employed (see Fig. 1). Rather than forward-scatter ones, the backscatter setup can be used to suppress significantly exponential decaying backgrounds induced by non-resonant processes, and hence to improve the signal to noise (S/N) ratio^28^. We run simulations on NRF $\gamma$-ray emission and detection of $^{238}$U at energies below 2.5 MeV. The irradiation of $^{238}$U target is considered as a representative scenario since the isotope $^{238}$U has several visible NRF peaks which have been observed in previous measurements^29,30^. The photon fluence *I*(*E*) in each simulation is composed of 10$^{11}$ photons with cut-off energy of 2.5 MeV, and is directed orthogonally at an isotopically-pure cylinder target without iron shielding. Note that such flux can be readily delivered by the state-of-the-art LCS $\gamma$-ray source within a few of seconds^31,32^. Figure 2a shows partial energy spectrum recorded by four HPGe detectors considering an achievable energy resolution of 0.05%. Seven NRF peaks are seen for $^{238}$U, from which these peak counts were extracted accordingly. Figure 2b compares Eq. (1) to the MC results for $^{238}$U targets of variable thickness *L*. For thicker target *L* $\ge$ 0.5 cm, the relative deviation between the simulations and the model predictions is better than 1 $\%$, which suggests the validation of our simulation algorithm.

### NRF signatures of illicit drugs

**Figure 3:**
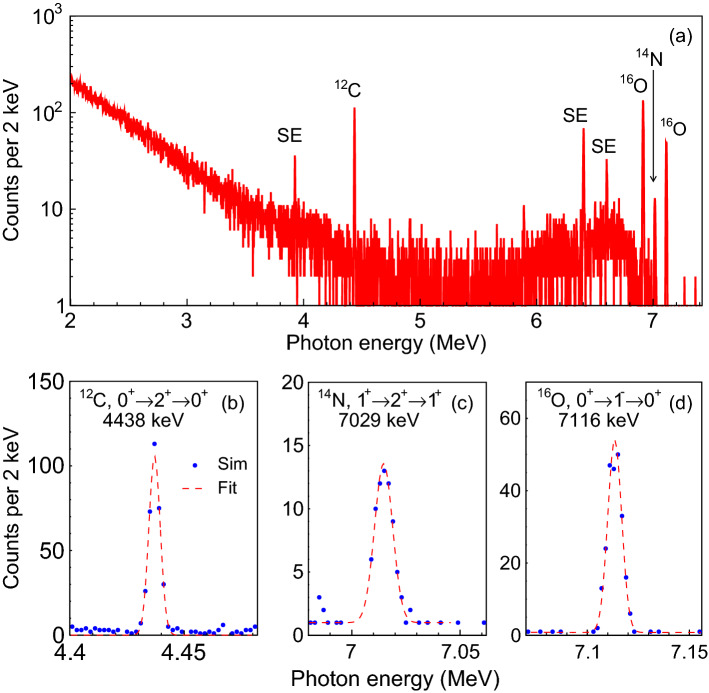
(**a**) Simulated NRF $\gamma$-ray spectra from the cocaine material without iron shielding. There are clear photopeaks at 4438, 6917, 7029 and 7116 keV at large S/N ratios, and three visible single-escape (SE) peaks. The cylinder target with a thickness of 10 cm, a radius of 5 cm and a density of 0.9 g/cm$^{3}$ and the $\gamma$-beam flux of 10$^{11}$ are used in the simulations. Three zoomed spectra showing the NRF signal regions around 4438 keV $^{12}$C (**b**), 7029 keV $^{14}$N (**c**) and 7116 keV $^{16}$O (**d**), respectively. In pads (**b**)–(**d**), dash lines represent 5-parameter Gaussian peak plus linear background fits when considering a narrow region of ± 40 keV.

Similarly, NRF signatures are obtained for five drug materials, cocaine, morphine, methamphetamine, ketamine and heroin. In the simulations, the cut-off energy of LCS $\gamma$-ray beam is shifted from 2.5 MeV to $\sim$ 7.2 MeV, which is obtained by the Compton backscattering of a 532 nm laser pulse from a 452 MeV electron beam with an energy spread of 0.05%. Regarding the MC simulation models and conditions, more detailed information is given in section of Methods. Figure 3 shows the exemplary energy spectrum for cocaine material, which is merged with four separate HPGe detectors to improve the S/N ratio. One can see four NRF signals can be clearly detected, where $^{12}$C contributes the 4438 keV (0$\rightarrow$2$\rightarrow$0) peak, $^{14}$N the 7029 keV (1$\rightarrow$2$\rightarrow$1) peak, and $^{16}$O the 6917 keV (0$\rightarrow$2$\rightarrow$0) and 7116 keV (0$\rightarrow$1$\rightarrow$0) peaks. Three NRF signal regions near 4438 keV $^{12}$C, 7029 keV $^{14}$N and 7116 keV $^{16}$O are then fitted separately with Gaussian function, on top of a quasi-linear decaying continuum background. The fitting function for each of these NRF peaks is written as

3 $$\begin{aligned} f(E) = c_{1} + c_{2} E + \frac{a}{\sqrt{2\pi } \sigma _{E}} exp\left[ -\frac{(E-E_{NRF})^2}{2 \sigma _{E}^{2}}\right] \end{aligned}$$

where $c_{1}$ and $c_{2}$ describe the shape of the background, and *a*, $E_{NRF}$ and $\sigma _{E}$ are the area, mean and standard deviation (SD) fit parameters of the peak. The fitting function can describe well the simulation curve (Fig. 3).

Once the 5-parameter fit (and associated parameter uncertainties) for each NRF peak is computed using Eq. (3), the detected NRF rate in counts were extracted as simply $Y = a/\Delta E$, where the division by the spectrum bin width $\Delta E$ enforces proper dimensions and normalization^9^. The peak uncertainty is calculated by $\delta Y=\sqrt{Y+2B}$ with *B* being the background yields. The NRF peak and background yields, and associated peak significance for five illicit drugs are summarized in Supplementary Section S1. Although the integration cross section is as low as 6.32 eV$\cdot$b, the NRF signal at 4438 keV for $^{12}$C can be detected with high confidence of 16−20$\sigma$ because of high mass fraction. For cocaine, heroin and morphine, the NRF lines at 6917 and 7116 keV of $^{16}$O exhibit comparable confidence (13−24$\sigma$) to the $^{12}$C. The NRF peak at 7029 keV for $^{14}$N has less production yield and then relatively low confidence ($\sim$ 7$\sigma$). This is mainly attributed to very small mass fraction while the integration cross section for the $^{14}$N is almost two times higher than the $^{12}$C. Moreover, the background level is almost one order of magnitude lower than the NRF signal. This is due to the non-resonant effect contributes visibly to the low energy region of the spectrum, given by that the LCS photon beam has sufficient intensity at the cutoff energy (7200 keV), which matches well to the 7116 keV $^{16}$O.

### Element ratio of illicit drugs

To further distinguish the types of illicit drugs, two-dimensional element ratio approach is proposed. After extracting the NRF peak yields, the element ratio $^{14}N/^{12}C$ can be obtained by

4 $$\begin{aligned} \frac{^{14}N}{^{12}C}=\frac{Y_{^{14}N}}{Y_{^{12}C}} \frac{I(E_{^{12}C})}{I(E_{^{14}N})} \frac{W_{^{12}C}(\theta )}{W_{^{14}N}(\theta )} \frac{\varepsilon (E_{^{12}C})}{\varepsilon (E_{^{14}N })} \frac{\int \sigma _{^{12}C}(E)dE}{\int \sigma _{^{14}N}(E)dE}, \end{aligned}$$

where the $Y_{^{14}N}/Y_{^{12}C}$ is the ratio between the $^{14}$N and $^{12}$C peak yields (see Supplementary Section S1). The intensity ratio ${I(E_{^{12}C})}/{I(E_{^{14}N})}$ and efficiency ratio ${\epsilon (E_{^{12}C})}/{\epsilon (E_{^{14}N})}$ were simulated with the code MCLCSS^33^, together with the Geant4 toolkit^34^. The ratios ${\int \sigma _{^{12}C}(E)dE}/{\int \sigma _{^{14}N}(E)dE}$ and ${W_{^{12}C}(\theta )}/{W_{^{14}N}(\theta )}$ are given by Eqs. (5) and (8), respectively. According to the rules of error propagation, the uncertainties associated with the predicted element ratios can be obtained by ${^{14}N}/{^{12}C}\sqrt{(\delta Y_{^{14}N}/Y_{^{14}N})^2+(\delta Y_{^{12}C}/Y_{^{12}C})^2}$, considering only the statistical uncertainties of peak yields. Similarly, one can obtain the ratio expression of ${N_{^{16}O}}/{N_{^{12}C}}$ and its uncertainty. Note that both the $^{16}O/^{12}C$ and $^{14}N/^{12}C$ are approximately identical to their respective isotopic ratios considering the isotopic abundances of $^{12}$C, $^{14}$N and $^{16}$O are almost 1.0.

**Figure 4:**
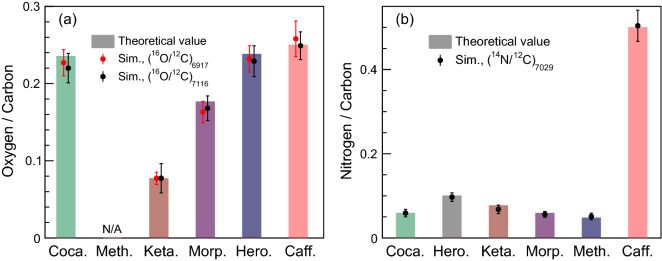
Element ratios of oxygen to carbon (**a**) and nitrogen to carbon (**b**). Coca., Meth., Keta., Hero. and Caff. are abbreviations for the drugs Cocaine, Methamphetamine, Ketamine, Heroin and Caffeine, respectively.

The resulting $^{16}O/^{12}C$ and $^{14}N/^{12}C$ for aforementioned five drug materials are shown in Fig. 4, together with their theoretical ratios as comparison. According to Eq. (4), the relative deviations of both the ratios $^{16}O/^{12}C$ and $^{14}N/^{12}C$ were calculated to be 0.4−11.6 $\%$. One can see that ketamine and morphine can be well distinguished from three other drugs when considering either the ratio $^{16}O/^{12}C$ or the $^{14}N/^{12}C$. Methamphetamine can be identified though the ratio of $^{14}N/^{12}C$ with the NRF peak at 7029 keV, since the ratio value is visibly higher than others. However, the ratio $^{16}O/^{12}C$ is not applicable for methamphetamine considering it has no oxygen content. For cocaine and heroin, they are almost identical regarding the ratio $^{16}O/^{12}C$ and have insignificant discrepancy (0.059:0.048) in the ratio $^{14}N/^{12}C$, which suggests that accurate discrimination of cocaine and heroin is challenging when the statistic is not sufficient.

It is also necessary to check the element ratio for benign materials that are not dissimilar to the various narcotics. Caffeine and related chemicals in coffee can be regarded as good candidates of benign materials since coffee is generally used by smugglers to overwhelm drug-smelling dogs^35^. We then performed such simulation by replacing the drug material inspected with caffeine (C$_{8}$H$_{10}$N$_{4}$O$_{2}$). The element ratios, $^{16}O/^{12}C$ and $^{14}N/^{12}C$, are calculated accordingly and compared to those of the five illicit drugs (see Fig. 4). The comparison shows the inspected drugs have significant difference in element ratios relative to the caffeine.

### Inspection of concealed cocaine

**Figure 5:**
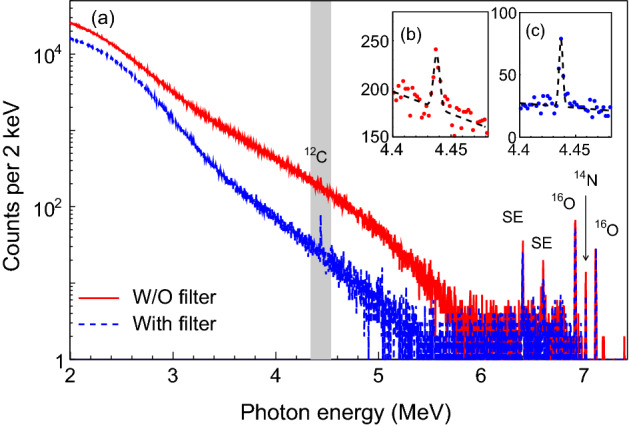
(**a**) Simulation result of energy spectrum merged from four HPGe detectors with / without 1-mm-thick lead filter. The insets show enlarged views of the shadow area: (**b**) and (**c**) are the the 4438-keV line and its fit curve for $^{12}$C in the absence and presence of lead filter, respectively. A 15-mm-thick iron material is used to conceal the drug target.

We have presented inspection of illicit drugs without any blocking. A more realistic scenario is to smuggle drugs in heavy concealment. Thus, it is of great importance to discuss the inspection feasibility on concealed drugs (see Fig. 1). Here we used 15-mm-thick iron to enclose drugs to be inspected such as cocaine. Such shielding reduces the photon intensity from 1.0 (relative) to 0.75 at $\sim$ 7 MeV, so it is for the NRF $\gamma$ rays emitted. Meanwhile, due to the $\gamma$-ray attenuation, a large number of backgrounds were produced. Figure 5 shows that the background photons produced in total are almost two orders of magnitude higher than without iron shielding (see Fig. 3). For cocaine, the peak significance at 4438, 6917, 7029 and 7116 keV are reduced from 16.7, 23.4, 7.7 and 15.6$\sigma$ to 3.5, 13.9, 3.6 and 9.1$\sigma$, respectively. In order to relieve the background effect on the signal detection, we introduced a 1-mm-thick lead filter in front of each HPGe detector. This reduces significantly the background for peak fitting at 4438 keV such that the NRF peak at 4438 keV $^{12}$C can be observed more clearly (see Fig. 5). This also provides a potential approach to enhance the S/N in the presence of metal shielding.

In order to demonstrate the feasibility to inspect drugs even when the illicit drugs are surrounded by benign materials containing C, H, O and N, we simulated the inspection of cocaine concealed in a wrapping material consist of caffeine (C$_{8}$H$_{10}$N$_{4}$O$_{2}$) with different areal densities. According to the simulated spectra, the NRF peak yields are extracted, and then the element ratios, $(^{16}O/^{12}C)_{6917}$, $(^{16}O/^{12}C)_{7116}$ and $(^{14}N/^{12}C)_{7029}$, and their associated uncertainties are obtained with the same manner described in the previous subsection. It is shown that the predicted element ratios, $(^{16}O/^{12}C)_{6917}$ and $(^{16}O/^{12}C)_{7116}$, stay almost the same as the areal density of caffeine grows (see Table 1). This is because caffeine has an element ratio $(^{16}O/^{12}C)$ of 0.25, which is very close to that of cocaine. One can see that when the areal density of caffeine is less than 0.30 g/cm$^{2}$, all predicted ratios are consistent with the theoretical values within the statistical uncertainty. As a result, the element ratio approach is still valid to identify drugs wrapped in thin materials containing C, H, O and N. However, for caffeine denser than 0.30 g/cm$^{2}$, the predicted ratio $(^{14}N/^{12}C)_{7029}$ increases rapidly due to the fact that the ratio of $(^{14}N/^{12}C)$ for caffeine is visibly larger than that of the cocaine.

**Table 1:** The predicted element ratio for cocaine wrapped in caffeine with different areal densities.

Areal density (g/cm$^{2}$)	$(^{14}N/^{12}C)_{7029}$	$(^{16}O/^{12}C)_{6917}$	$(^{16}O/^{12}C)_{7116}$
0.00	0.059 ± 0.008	0.227 ± 0.016	0.220 ± 0.018
0.15	0.057 ± 0.008	0.222 ± 0.016	0.219 ± 0.022
0.30	0.073 ± 0.009	0.218 ± 0.016	0.220 ± 0.022
0.75	0.157 ± 0.014	0.228 ± 0.016	0.229 ± 0.020
Theoretical value	0.059	0.235	0.235

## Discussion

We have proposed a novel inspection method for achieving drug components and composition, which can be used to obtain a unique fingerprint of the drug. The proposed method is based on NRF spectroscopy plus element ratio approach, benefiting from an intense LCS $\gamma$-ray source. MC simulations show that NRF signatures of the drug components, $^{12}$C , $^{14}$N and $^{16}$O, can be detected with a high confidence of 7−24$\sigma$ with a $\gamma$-ray flux of 10$^{11}$. The element ratios, $^{16}O/^{12}C$ and $^{14}N/^{12}C$, for five widely abused drugs are then identical with the exact ratios within relative deviation of 11.6 $\%$.

We further evaluate systematic uncertainty in element ratios predicted with Eq. (4), in view of uncertainty of NRF cross sections, instability of LCS $\gamma$-ray beam and unavailability of angular correlation $W(\theta )$ (see Section S3 of Supplementary materials). First, the predicted elemental ratios, $(^{16}O/^{12}C)_{6917}$, $(^{16}O/^{12}C)_{7116}$ and $(^{14}N/^{12}C)_{7029}$, have respectively uncertainties of 6.9%, 7.8% and 13.5% due to the uncertainty in NRF cross sections. Second, the instability of the LCS beam has invisible impacts on the element ratio predictions because of the intrinsic synchronous fluctuation of photon intensity at different photon energies. Third, the NRF lines at 4438 keV ($^{12}$C), and 6917 keV ($^{16}$O) and 7116 keV ($^{16}$O) have a purely quadrupole-quadrupole or dipole-dipole transition and the resulting $W(\theta )$ have exact expressions, which will not lead to observable uncertainties in element ratios. However, an exact expression of $W(\theta )$ is not available for the NRF line at 7029 keV ($^{14}$N), due to the unavailability of mixing ratio. Currently it is very hard to evaluate the effect of uncertainty in $W(\theta )$ on element ratio. Here we call for both experimental and theoretical efforts towards the accurate determination of real values of $W(\theta )$. Moreover, the element ratio approach can hardly be used to identify benign materials with very closed element concentrations to the drug materials inspected, considering that the predicted element ratios would be the same as those of the drug materials. In this situation, the inspector should be alerted to take further actions, such as chemical identification with the Raman spectroscopy.

**Figure 6:**
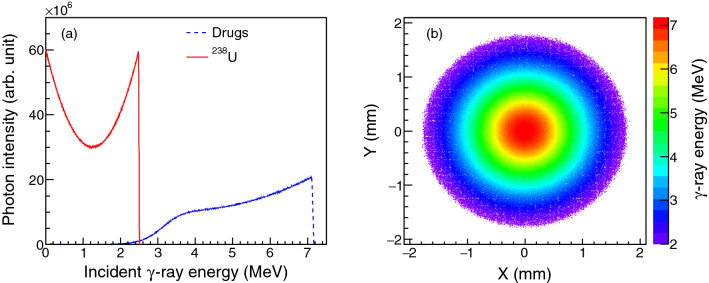
(**a**) Spectral distributions of LCS $\gamma$-ray beams incident upon the $^{238}$U and drug targets. (**b**) Spatial distribution of LCS $\gamma$-ray beam incident upon the drug targets.

We should note that the $\gamma$-ray beam can probe different parts of inspected material due to the spatial non-uniformity, which makes NRF $\gamma$ rays emitting from different positions of the target and then induces uncertainty to the simulated NRF yields. In our simulations, such uncertainty has been included in the relative deviations of the element ratios predicted. When the spot size of the $\gamma$-ray beam incident upon the target (see Fig. 6) is far smaller than the target dimension (see the caption of Fig. 3), it can be regarded as a kind of point-like $\gamma$-ray source with good directionality. Then the spatial non-uniformity of such a beam would not impact on the fidelity of the element ratio predictions. In a realistic setup, in case the beam size is comparable to the target dimension, one should address this issue carefully.

The detectable drug mass can reach gram level, given by that the target size can be shrank from 5 cm (see caption of Fig. 3) to a few mm, which is comparable to the size of LCS $\gamma$-ray beam produced by hundreds of MeV electron beam^25^. Note that such small target would not reduce the NRF yields but benefit the NRF $\gamma$-ray transportation to the HPGe detectors. These results suggest that the NRF spectroscopy combined with the element ratio approach will enable us to identify drugs in a portable package within a practical measurement time. Our proposed method can still be extended to screen containers and identify SNM relevant to weapon of mass destruction and hazardous chemical compounds like explosives. Recently, compact X/$\gamma$-ray source generators have been developed^36–40^, which may offer great opportunity to adopt the proposed method to seaports and national borders.

## Methods

### NRF $\gamma$ rays

NRF refers to nuclear resonant absorption of a $\gamma$ photon followed by the de-excitation with emission of $\gamma$ rays. The energies of the states excited by NRF is inherent in the atomic number and the mass of the nucleus of interest (see Fig. 1b). The NRF cross section for absorption via the resonant energy level $E_{r}$ is given by Breit-Wigner distribution^41^:

5 $$\begin{aligned} \sigma _{NRF}(E)=\frac{g}{2}\pi \frac{(\hbar {c})^2}{E_{r}^2}\frac{\Gamma \Gamma _{0}}{(E-E_{r})^2+(\Gamma /2)^2}, \end{aligned}$$

where $\Gamma$ is the width of the level at $E_{r}$, $\Gamma _{0}$ is the partial width for transitions between $E_{r}$ and the ground state, $\hbar$ is the Plank constant, *c* is speed of light.

In practise, the NRF cross section should be calculated with taking Doppler broadening into account. Approximating the true Voigt profile to a Gaussian profile, Eq. (5) is then transformed into^42^:

6 $$\begin{aligned} \sigma _{D}(E)\approx (\frac{\hbar c}{E_{r}})^{2}\frac{\pi ^{3/2}}{\sqrt{2}\Delta }g \frac{\Gamma _{0}^{2}}{\Gamma }exp(\frac{(E-E_{r})^2}{2\Delta ^2}). \end{aligned}$$

Here $\Delta$ = $E_{r}\sqrt{{k_{B}T}/{Mc^2}}$ is the Doppler width, $k_{B}$ is the Boltzman constant, *T* is the absolute temperature, and *M* is the mass number of the nucleus. For low-*Z* isotopes of interest, their fundamental widths are typically $\sim$ 10 meV. The effective width of the cross section after Doppler broadening increases to $\sim$ 10 eV. However, considering that in our case the irradiation targets exist in a molecular form, *M* should be replaced with the mass number of the molecule. As a consequence, the Doppler widths decrease significantly by a factor of $\sim$ 5. Imperfect detector resolution further broadens the detectable NRF resolution to widths of about 2–3.5 keV (see Fig. 3). The NRF cross sections of $^{12}$C, $^{14}$N and $^{16}$O are discussed in Supplementary Section S2.

Due to conservation of energy and momentum, a free nucleus undergoing NRF will recoil with kinetic energy $E_{rec}$ determined by the Compton-like formula:

7 $$\begin{aligned} E_{rec}=E_{r} \left[ 1 - \frac{1}{1 + E_{r}(1 - cos \theta )/Mc^{2})}\right] \approx \frac{E_{r}^2}{2Mc^2} (1 - cos \theta ), \end{aligned}$$

where $\theta$ is photon scattering angle relative to its incident direction. It is worth to note that the recoil energy for the drug molecules is $\sim$ 200 eV, while the width of the Doppler-broaden NRF interaction is $\sim$ 2 eV. Therein, the energy of the emitting photons are too small to trigger another NRF interaction of the same transition.

NRF is generally considered to occur only between states that differ by two or fewer units of angular momentum. The angular distribution of NRF $\gamma$ rays is analogous to that of $\gamma$-ray cascades. For an NRF interaction of transitions $J_{a}(L_{1})J_{b}(L_{2})J_{c}$, with $L_{1}$ and $L_{2}$ being the multipole orders of the excitation and de-excitation, respectively, the angular distribution $W(\theta )$ can be written as^43^:

8 $$\begin{aligned} W(\theta )=1+A_{2}P_{2}(cos\theta )+\cdots +A_{2n}P_{2n}(cos\theta ), \end{aligned}$$

where $P_{2n}(cos\theta )$ is the Legendre polynomial expansion, and $A_{2n}$ is given by:

9 $$\begin{aligned} A_{2n}=F_{2n}(L_{1}J_{a}J_{b}) F_{2n}(L_{2}J_{c}J_{b}). \end{aligned}$$

where $F_{2n}(L_{1}J_{a}J_{b})$ and $F_{2n}(L_{2}J_{c}J_{b})$ are two constants depending on spin states of transitions and photon multipolarities^44^. The angular correlations of NRF $\gamma$ rays from $^{12}$C, $^{14}$N and $^{16}$O are discussed in Supplementary Section S3.

### MC simulations

In this study, LCS $\gamma$-ray beam was produced by Compton backscattering of 532-nm-wavelength laser pulse with high-energy electron beam. The generation of the LCS $\gamma$-ray beam was simulated with the code MCLCSS^33^. After generation, the delivery, transportation and/or collimation of the LCS $\gamma$ rays were performed with Geant4 toolkit.

To model NRF interactions, we developed a new class G4NRF into the Geant4 toolkit^34,45^. The pure virtual method G4VUserPhysicslist::ConstrcutProcess() was implemented in the simulation, and the method AddDiscreteProcess() was used for registration of the newly developed physics process. Customizing NRF process into the simulation requires the implementation of two features: first, to provide the cross sections for interaction and second, to determine the final state resulting from the interaction. The calculation of a series of NRF cross sections was given by Eq. (6). The information of final states is provided by Eq. (8).

In all simulations, the root-mean-square (RMS) value of the electron energy spread of electron is fixed to 0.05%. The normalized electron emittance is $\epsilon _{n}$ = 0.2 mm$\cdot$mrad and the RMS beam size at the interaction point is $\sigma _{e}$ = 0.03 mm. In the scenario of $^{238}$U irradiation, a 266-MeV electron beam was employed to generate the LCS $\gamma$-ray beam with cut-off energy of 2.5 MeV. Such un-collimated $\gamma$-ray beam was then incident upon a cylindrical $^{238}$U target with a radius of 5.0 cm. In the scenario of drug inspection, the energy of electron beam was adjusted to 452 MeV, such that the $\gamma$-ray beam generated reaches a cut-off energy of $\sim$7.2 MeV. In addition, a collimator with collimation angle of 1.5 mrad was employed to avoid the drug material irradiated by LCS photons at low energies, and the beam spot size on the target surface is several mm. In this scenario, the cocaine, heroin, ketamine, methamphetamine and morphine targets are also cylindrical ones with radius of 5.0 cm and areal density of 9.0 g/cm$^{2}$. Their compositions are specified to be C$_{17}$H$_{21}$NO$_{4}$, C$_{21}$H$_{23}$NO$_{5}$, C$_{13}$H$_{16}$ONCl, C$_{10}$H$_{15}$N and C$_{17}$H$_{19}$NO$_{3}$, respectively. The spectral and spatial distributions of the LCS $\gamma$-ray bemas used for these target irradiations are shown in Fig. 6.

The backscatter NRF photons resulted from irradiation targets are recorded by four HPGe detectors assembled at 135$^{\circ }$ relative to the LCS beam direction (see Fig. 1). It is supposed that the HPGe detectors have an energy resolution of 0.05% (in RMS), which is achievable with the present detector technology^46,47^. The Ge crystals have a 10 cm diameter and a 10 cm length. The full-energy peak efficiency of each HPGe detector, $\epsilon (E)$, is simulated with the Geant4 toolkit. Accordingly, the $\epsilon (E)$ at 4438, 7029, 6917 and 7116 keV are extracted in order to perform element ratio predictions. Four HPGe detectors are shielded by a 1.0-mm-thick lead plate, avoiding a large part of low-energy photons entering into the HPGe detector.

## Supplementary information

Supplementary Information

## Data Availability

The data that support the plots within this article and other findings of this study are available from the corresponding authors on reasonable request.
