# Supplementary material for: Nuclear resonance fluorescence drug inspection
Source: Sci Rep. 2021 Jan 14;11:1306. doi: 10.1038/s41598-020-80079-6 (PMC7809066; doi:10.1038/s41598-020-80079-6)
Supplement: Supplementary file 1 — Supplementary Information [file 41598_2020_80079_MOESM1_ESM.docx]

# Supplementary material for

# Nuclear resonance fluorescence drug inspection

Haoyang Lan, Tan Song, Xingde Huang, Shengqiang Zhao, Jianliang Zhou, Zhichao Zhu, Yi Xu, Dimiter L. Balabanski, and Wen Luo

## Section S1. Extraction of NRF signature

For five drug materials, NRF signature are extracted by fitting the energy spectra recorded by HPGe detectors. The extracted NRF signatures are summarized in Table S1. Given by Eq. 3, the peak yields are calculated by *Y*=*a/*Δ*E* with Δ*E* being the spectrum bin width*.* Accordingly, the background yields *B* are obtained by integrating (*c_1_*+*c_2_E*)*/*Δ*E* over a fixed interval of *E_NRF_* ± 3*σ_E_*. The peak uncertainty is calculated by $\delta Y=\sqrt{Y+2B}$. The resulting peak significance is given by $Y/\delta Y$.

**Table S1.** Fitting results of simulated NRF γ-ray spectra associated with five drug materials.

| Chemical compound | Energy level | Peak yield | Background | Statistical significance (*σ*) |
| --- | --- | --- | --- | --- |
| Cocaine  C_17_H_21_NO_4_ | 4438 keV (^12^C) | 311 | 19 | 16.7 |
|  | 7029 keV (^14^N) | 76 | 11 | 7.7 |
|  | 6917 keV (^16^O) | 592 | 23 | 23.4 |
|  | 7116 keV (^16^O) | 268 | 14 | 15.6 |
| Heroin  C_21_H_23_NO_5_ | 4438 keV (^12^C) | 320 | 20 | 16.9 |
|  | 7029 keV (^14^N) | 64 | 11 | 6.9 |
|  | 6917 keV (^16^O) | 623 | 25 | 24.0 |
|  | 7116 keV (^16^O) | 287 | 18 | 16.0 |
| Ketamine C_13_H_16_ONCl | 4438 keV (^12^C) | 297 | 22 | 16.1 |
|  | 7029 keV (^14^N) | 79 | 11 | 7.9 |
|  | 6917 keV (^16^O) | 192 | 12 | 13.1 |
|  | 7116 keV (^16^O) | 90 | 22 | 7.9 |
| Methamphetamine C_10_H_15_N | 4438 keV (^12^C) | 405 | 15 | 19.4 |
|  | 7029 keV (^14^N) | 154 | 0 | 12.4 |
|  | 6917 keV (^16^O) | N/A | N/A | N/A |
|  | 7116 keV (^16^O) | N/A | N/A | N/A |
| Morphine C_17_H_19_NO_3_ | 4438 keV (^12^C) | 323 | 23 | 16.8 |
|  | 7029 keV (^14^N) | 71 | 11 | 7.4 |
|  | 6917 keV (^16^O) | 442 | 16 | 20.3 |
|  | 7116 keV (^16^O) | 212 | 16 | 13.6 |

## Section S2. Calculation of NRF cross section

## The NRF reaction cross sections for ^12^C, ^14^N and ^16^O below 7.2 MeV is shown in Fig. S[1](#bookmark0). These NRF lines are very slim due to their extremely narrow Doppler width Δ, of the order of 10 eV. According to Eq. 6, one can further obtain the integrated NRF cross sections, *σ*_int_ (see Table S2). The level widths used for cross section calculations are obtained from national nuclear data center (NNDC) [S1]. Note that the experimental data of *σ*_int_ for ^12^C, ^14^N and ^16^O is not available. As a result, the calculated NRF cross sections were implemented into the Geant4 to describe the NRF processes. To validate the calculation of NRF cross sections for ^12^C, ^14^N and ^16^O, we compare the calculated *σ*_int_ to the experimental ones for two principal NRF lines of ^238^U at 1782 and 1846 keV [S2]. It is found that the calculation is in good agreement with the experimental data.


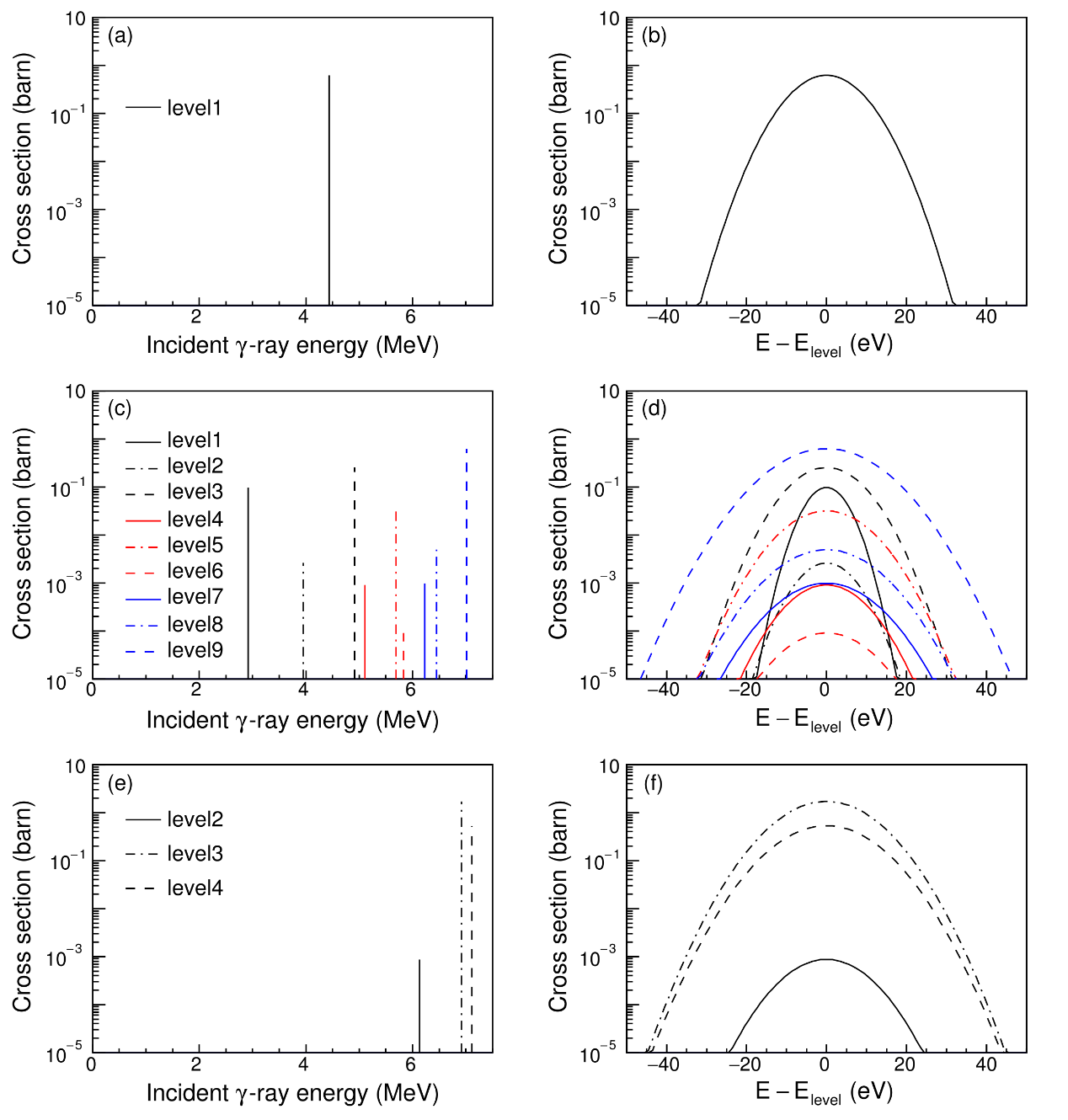


**Fig. S1.** (a), (c) and (e) are the cross section calculated by Eq. 6 for different NRF lines of ^12^C, ^14^N and ^16^O, respectively. (b), (d) and (f) are the NRF reaction cross section as a function of E −Elevel for different reaction channel of ^12^C, ^14^N and ^16^O, respectively. Elevel is the level energy of different excited states. In the calculations, a room temperature of *T* = 300 K is considered.

The reasons to choose the NRF lines of ^12^C, ^14^N and ^16^O used for drug inspections are as follows. There is only one available NRF line (at 4438 keV) for ^12^C. The *σ*_int_ is obtained to be 6.32 ± 0.40 eV·b accordingly (see Table S2). For ^14^N, the NRF channel at 7029 keV is selected since the value of *σ*_int_ reaches 15.58 ± 1.68 eV·b, which is four times higher than the one at 4915 keV. For ^16^O, the NRF cross section of the first excited state is not shown because the spin of the first excited state is the same as that of the ground state, which is zero. The γ transition between these two states is forbidden since photon itself carries an angular momentum of 1, according to the selection rules of multipole transitions. For the rest of NRF lines, both the ones at 6917 keV and 7116 keV, carrying considerable *σ*_int_, are suitable for the identification of ^16^O.

**Table S2.** Nuclear excitation levels of ^12^C, ^14^N, ^16^O and ^238^U associated with energy levels, widths, and the integrated NRF cross sections.

| Isotope | *E_γ_* (keV) | *Γ* (meV**)** [S1] | *σ_int_* (b·eV) |
| --- | --- | --- | --- |
| ^12^C | 4438 | 10.8 ± 0.6 | 6.32 ± 0.40 |
| ^14^N | 4915 | 84 ± 16 | 4.43 ± 0.83 |
|  | 7029 | 123.3 ± 13.3 | 15.58 ± 1.68 |
| ^16^O | 6917 | 97.07 ± 2.68 | 38.95 ± 1.08 |
|  | 7116 | 54.9 ± 3.3 | 12.49 ± 0.75 |
| ^238^U | 1782 | 13.80 ± 1.03 | 20.90 ± 1.56 |
|  | 1846 | 14.71 ± 1.89 | 21.80 ± 2.81 |

## Section S3. Angular distribution of NRF photons

Eq. 8 indicates that the angular distribution of NRF γ rays is fully determined by both the spins of the states involved in an NRF interaction and the multipolarities of both the incident and emitting γ rays. The ground state spins of ^12^C, ^14^N and ^16^O are 0, 1 and 0, respectively. Thus, transitions of the forms such as 0→1→0, 0→2→0, 1→0→1, 1→1→1, 1→2→1 and 1→3→1 are all expected to be possible.

The unnormalized angular distributions for NRF photons of all these transitions are depicted as polar diagrams in Fig. S2. One can see that in the case of unpolarized photon beams, the angular patterns of purely dipole-dipole transitions are in elongated elliptical shapes, and the patterns of purely quadrupole-quadrupole transitions are in cloverleaf shapes. For transitions where a spin 0 state is involved, such as 0→1→0, 0→2→0, 1→0→1, only a single photon multipolarity is allowed. For 0→1→0 transitions (only dipole transitions allowed), the angular correlation function is *W*(*θ*) = 0.75(1 + cos2*θ*) and for 0→2→0 transitions (only quadrupole transitions allowed), it is *W*(*θ*) = 1.25(1 − 3cos^2^*θ* + 4cos^4^*θ*). These two angular correlation functions were widely adopted by previous studies, such as Ref. [4] and Ref. [15]. For transitions between states that exclude spin 0 states, i.e, 1→1→1, 1→2→1 and 1→3→1, the description of *W*(*θ*) becomes more complicated since they may include more than one multipolarity. The exemplary angular correlation functions for 1→2→1 in different multipolarity configurations are shown in Fig. S3. One can see that the *W*(*θ*) for dipole-quadrupole and quadrupole-dipole transitions are the same. Meanwhile, the purely-dipole and purely-quadrupole ones show significant differences. According to spin rules, all these four multipolarity combinations are allowed. The actual *W*(*θ*) is hard to be deduced due to the unavailability of mixing ratio data. Similarly, the actual *W*(*θ*) is still unknown for 1→1→1 and 1→3→1 since the corresponding mixing ratios are not yet determined. Consequently, we simply assume a purely quadrupole-quadrupole transition for 1→2→1 and 1→3→1, and a purely dipole-dipole transition for 1→1→1.


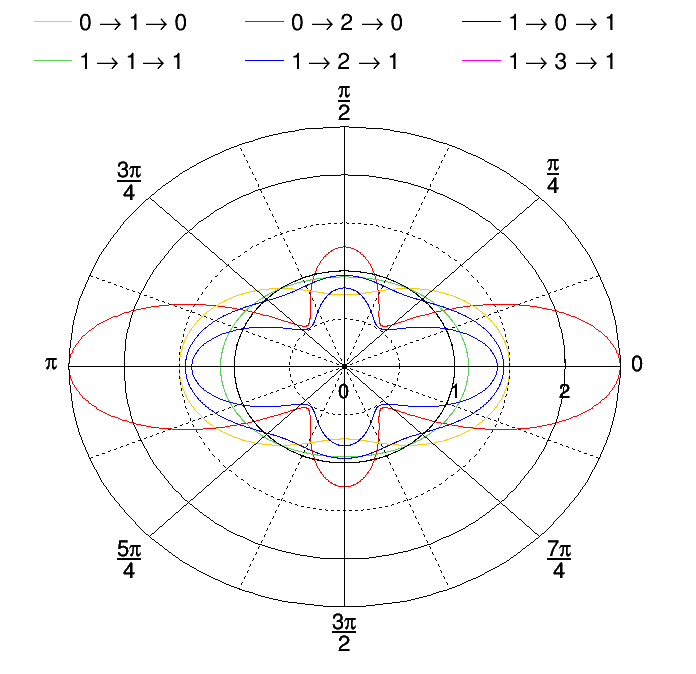


**Fig. S2.** The angular correlation functions for NRF between states of initial and final spins 0 and 1 that allowed by dipole and quadrupole radiations.


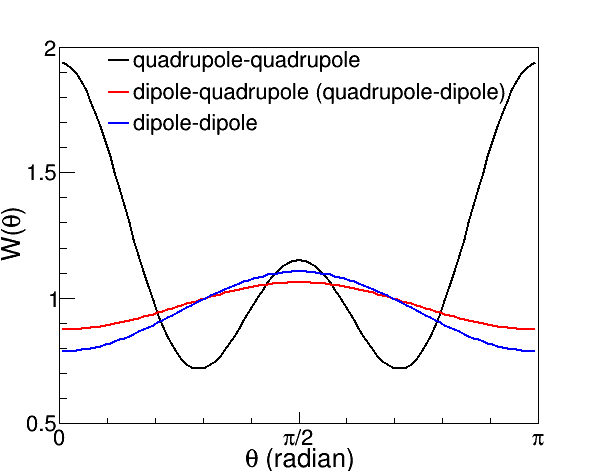


**Fig. S3.** The angular correlation functions of transition 1→2→1 for three hypothetical multipolarity combinations: only dipole (blue line), only quadrupole (black line), and one transition dipole, the other quadrupole (red line).

## Section S4. Systematic uncertainty in element ratios

We evaluate systematic uncertainty in element ratios predicted with Eq. 4, in view of uncertainty of NRF cross sections, instability of LCS γ-ray beam and indetermination of angular correlation *W*(*θ*).

Firstly, the uncertainty of NRF cross sections will affect the accuracy of predicted elemental ratio. The *σ*_int_ can be written as

$$\sigma_{int}=\int\sigma_{NRF}\left( E \right)dE\approx\pi^{2}\frac{2J_{i}+1}{2J_{0}+1}{(\frac{\hbar c}{E_{\gamma}})}^{2}\Gamma$$

Here, *J_i_* and *J*_0_ are the spin of the resonant state and the ground state of the target nucleus, and *Γ* is the level width at resonant energy *E_γ_*. It is shown that the uncertainty of *σ*_int_ is proportional to that of *Γ* since those uncertainties induced by other variables can be ignored. One can obtain readily the widths *Γ* for ^12^C, ^14^N and ^16^O, and their uncertainties from the evaluated nuclear structure data file (ENSDF) [S2]. Table S2 summarizes the resonant energy, level widths, and integrated NRF cross sections for ^12^C, ^14^N and ^16^O. It is shown that the uncertainties for level width and NRF cross section are less than ~11%. According to uncertainty propagation, such uncertainties can result in significant uncertainties of 6.9%, 7.8% and 13.5%, respectively, for the elemental ratios of (^16^*O/*^12^*C*) _6917_, (^16^*O/*^12^*C*) _7116_ and (^14^*N/*^12^*C*) _7029_.

Secondly, we analyzed the instability of the LCS beam, which include the γ-ray flux variation and the spectral broadening. The intensity ratios, such as $I(E_{.^{12}C})/I(E_{.^{14}N})$, are not dependent on γ-ray intensity variation because of the intrinsic synchronous fluctuation of the photon intensities at different energies. Spectral broadening is mainly resulted from the energy spread of electron beam interacting with laser pulse. In the simulations, we fixed the energy spread of electron beam to be 0.05%. As the electron energy spread is increased to 0.5%, the result shows that the intensity ratio $I(4438)/I(7116)$ is influenced by the increased electron energy broadening. However, the intensity ratios remain unchanged, if the cut-off energy of the γ-ray beam is visibly higher than the maximal *E_r_* used for element ratio predictions.

Thirdly, we further discuss the indetermination of angular correlation *W*(*θ*), which may affect the element ratio predictions. For the NRF interactions at 4438 keV (^12^C) and 6917 keV (^16^O), they both take a form of 0→2→0, in which only a purely quadrupole-quadrupole transition is theoretically permitted. Similarly, the NRF at 7116 keV (^16^O) is of the form 0→1→0, in which a purely dipole-dipole transition is considered. As a result, these angular correlations will not induce additional systematic uncertainty in the element ratios.

However, when the spin rules permit multiple types of transitions, the resulting angular correlation becomes undeterminable, as discussed above. For instance, the NRF interaction on ^14^N at 7029 keV follows a transition sequence of 1→2→1. According to spin rules, both the excitation and de-excitation would occur via quadrupole or dipole transitions. Because the relative intensity of transition multipolarities for such transition is not yet determined, we simply assume a purely quadrupole-quadrupole transition. However, due to the unavailability of mixing ratio for NRF line at 7029 keV (^14^N), currently it is very hard to evaluate the effect of uncertainty in *W*(*θ*) on element ratios. We sincerely call for both experimental and theoretical efforts towards the accurate determination of real values of *W*(*θ*).

**References**

[S1] http://www.nndc.bnl.gov/ensarchivals/

[S2] Zilges, A., et al. "Strong dipole excitations around 1.8 MeV in ^238^U." *Physical Review C* 52.2 (1995): R468.
